# Molecular Tumor Board of the University Medical Center Groningen (UMCG-MTB): outcome of patients with rare or complex mutational profiles receiving MTB-advised targeted therapy

**DOI:** 10.1016/j.esmoop.2024.103966

**Published:** 2024-11-04

**Authors:** V.D. de Jager, P. Plomp, M.S. Paats, S. van Helvert, A.ter Elst, A. van den Berg, H.J. Dubbink, W.H. van Geffen, L. Zhang, L.E.L. Hendriks, T.J.N. Hiltermann, B.I. Hiddinga, L.B.M. Hijmering-Kappelle, M. Jalving, J. Kluiver, B. Koopman, M. van Kruchten, E.M.J. van der Logt, B. Piet, J. van Putten, B.H. Reitsma, S.R. Rutgers, M. de Vries, J.A. Stigt, M.R. Groves, W. Timens, S.M. Willems, L.C. van Kempen, E. Schuuring, A.J. van der Wekken

**Affiliations:** 1 Department of Pathology and Medical Biology, University of Groningen, University Medical Center Groningen, Groningen, the Netherlands; 2 Department of Respiratory Medicine, Isala Hospital, Zwolle, the Netherlands; 3 Department of Pulmonary Medicine, Erasmus MC Cancer Institute, University Medical Center Rotterdam, Rotterdam, the Netherlands; 4 Department of Pathology, Radboud University Medical Center, Nijmegen, the Netherlands; 5 Department of Pathology, Erasmus MC Cancer Institute, University Medical Center Rotterdam, Rotterdam, the Netherlands; 6 Department of Pulmonary Diseases, Medical Center Leeuwarden, Leeuwarden, the Netherlands; 7 Structural Biology in Drug Design, Groningen Research Institute of Pharmacy, University of Groningen, Groningen, the Netherlands; 8 Department of Pulmonary Diseases, GROW – School for Oncology and Reproduction, Maastricht University Medical Center, Maastricht, the Netherlands; 9 Department of Pulmonary Diseases and Tuberculosis, University of Groningen, University Medical Center Groningen, Groningen, the Netherlands; 10 Department of Medical Oncology, University of Groningen, University Medical Center Groningen, Groningen, the Netherlands; 11 Department of Molecular Diagnostics, Pathology Friesland, Leeuwarden, the Netherlands; 12 Department of Pulmonary Diseases, Radboud University Medical Center, Nijmegen, the Netherlands; 13 Department of Pulmonary Diseases, Martini Hospital, Groningen, the Netherlands; 14 Department of Pulmonology, Nij Smellinghe, Drachten, the Netherlands; 15 Department of Pulmonology, Treant Hospital Group, Scheper Hospital, Emmen, the Netherlands; 16 Department of Pulmonology, Tjongerschans, Heerenveen, the Netherlands

**Keywords:** molecular tumor board, targeted therapy, precision oncology, real-world data, clinical decision making, molecular pathology

## Abstract

**Purpose:**

Molecular tumor boards (MTBs) are considered beneficial for treatment decision making for patients with cancer with uncommon, rare, or complex mutational profiles. The lack of international MTB guidelines results in significant variation in practices and recommendations. Therefore, periodic follow-up is necessary to assess and govern MTB functioning. The objective of this study was to determine the effectiveness of MTB treatment recommendations for patients with rare and complex mutational profiles as implemented in the MTB of the University Medical Center Groningen (UMCG-MTB) in 2019-2020.

**Patients and methods:**

A retrospective follow-up study was carried out to determine the clinical outcome of patients with uncommon or rare (combinations of) molecular aberrations for whom targeted therapy was recommended as the next line of treatment by the UMCG-MTB in 2019 and 2020.

**Results:**

The UMCG-MTB recommended targeted therapy as the next line of treatment in 132 of 327 patients: 37 in clinical trials, 67 in the on-label setting, and 28 in the off-label setting. For on- and off-label treatment recommendations, congruence of recommended and received treatment was 85% in patients with available follow-up (67/79). Treatment with on-label therapy resulted in a response rate of 50% (21/42), a median progression-free survival (PFS) of 6.3 months [interquartile range (IQR) 2.9-14.9 months], and median overall survival (OS) of 15.8 months (IQR 6.4-34.2 months). Treatment with off-label therapy resulted in a response rate of 53% (8/15), a median PFS of 5.1 months (IQR 1.9-7.3 months), and a median OS of 17.7 months (IQR 5.1-23.7 months).

**Conclusion:**

Treatment with MTB-recommended next-line targeted therapy for patients with often heavily pretreated cancer with rare and complex mutational profiles resulted in positive overall responses in over half of patients. Off-label use of targeted therapies, for which there is sufficient rationale as determined by an MTB, is an effective treatment strategy. This study underlines the relevance of discussing patients with rare and complex mutational profiles in an MTB.

## Highlights

• We describe the outcome of MTB-advised targeted therapy for patients with rare or complex mutations.
• MTB-advised targeted therapy was received by the vast majority of patients.
• Treatment with MTB-advised targeted therapy resulted in positive responses in over half of the patients.
• MTB-advised off-label prescription of targeted therapy was an effective treatment strategy.
• Patients with cancer harboring rare and complex mutations should be discussed in an MTB.

## Introduction

Since the introduction of next-generation sequencing (NGS) for detecting genomic alterations in different tumor types, a growing number of medical centers have instated multidisciplinary molecular tumor boards (MTBs). These MTBs interpret molecular tumor profiles for which current guidelines provide insufficient support (e.g. certain rare or complex mutations) and formulate recommendations for potential (targeted) therapies.^1^, ^2^, ^3^, ^4^, ^5^ However, international guidelines for the composition of MTBs, case selection, and decision making for treatment recommendations are lacking. This may result in heterogeneity in patient populations discussed at MTBs and variability in treatment recommendations that make evaluation of the effectiveness of MTBs challenging. A systematic review by Larson et al.^6^ has not only indicated the putative beneficial effect of MTBs, but also showed the variety in the characteristics and outcomes of MTBs in different countries. Examples are differences in case selection, congruence of recommended and received treatment, and response rates. It is therefore imperative that MTBs are periodically evaluated for advice adherence and treatment outcome.

Current international treatment guidelines for various (sub)types of cancer contain targeted therapies for certain molecular aberrations, with a prominent role of targeted therapy in guidelines for non-small-cell lung cancer (NSCLC). In 2018, 13 Food and Drug Administration (FDA)-approved targeted therapies were available for actionable mutations found in five different genes in NSCLC.^7^ In 2021, this number had doubled to 26 FDA-approved targeted therapies for mutations found in 10 different genes in NSCLC.^8^ In the 2023 Clinical Practice Guideline for metastatic NSCLC of the European Society of Medical Oncology (ESMO), predictive testing is recommended for biomarkers in 11 genes (*EGFR*, *KRAS* G12C, *BRAF* V600, *MET* exon 14 skipping, *ERBB2*, *ALK*, *ROS1*, *RET*, *NTRK1/2/3*) and for each biomarker at least one targeted therapy is recommended in the first or second line of therapy.^9^ Similarly, in other tumor types, such as melanoma, breast cancer, and colon cancer, (national) guidelines include indications for the use of targeted therapies in patients with specific molecular aberrations, such as *BRAF* V600 mutations in melanoma and colon cancer, and *PIK3CA* mutations in breast cancer.^10^^,^^11^ In the MTB of the University Medical Center Groningen (UMCG-MTB), patients are only reviewed for targeted therapy eligibility in case of complex or rare mutational profiles, defined as current national treatment guidelines providing insufficient guidance with regard to the preferred targeted therapy approach.^12^^,^^13^ Predominantly discussing complex or rare molecular profiles of routine biomarker testing, the vast majority of cases reviewed by the UMCG-MTB concern patients with an advanced-stage pulmonary malignancy.

As approvals for new targeted therapies for clinical use are constantly being updated and incorporated into treatment guidelines for standard-of-care practice, the molecular tumor profiles that need to be reviewed in an MTB are continuously changing. Furthermore, newly available therapies targeting mutation(s) previously considered to be unactionable settings may improve clinical outcomes of patients with cancer.^14^ These rapid developments impact the characteristics and molecular tumor profiles of patients enlisted for and reviewed by MTBs.

The UMCG-MTB has previously reported adherence to and effectiveness of treatment recommendations for patients with NSCLC in 2018.^13^ In this study, we present a retrospective follow-up analysis on the adherence to MTB advice and clinical outcome of patients with cancer reviewed by the UMCG-MTB in 2019 and 2020. Hereby, this study contributes to the further optimization and standardization of the treatment of patients, particularly for patients with advanced-stage lung cancer.

## Methods

The workflow of the UMCG-MTB has previously been described in detail by Koopman et al.^13^ In short, UMCG-MTB meetings occur weekly and are attended by pulmonary and medical oncologists, pathologists, clinical scientists in molecular pathology structural biologists, and upon request a clinical geneticist. Functioning as a regional MTB, clinical cases can be submitted for MTB review by both healthcare professionals of the UMCG and external centers. The UMCG-MTB functions as MTB for all nonacademic centers in four provinces of the Netherlands (Groningen, Drenthe, Friesland, and Overijssel) with a combined population of 2.9 million people. In addition, centers or MTBs from other regions may submit clinical cases for review by the UMCG-MTB.

MTB review is requested at the discretion of the requesting healthcare professional. If national guidelines provide adequate information for treatment, patients are not discussed in the UMCG-MTB. As described previously, molecular 3D modeling is used by the UMCG-MTB to provide (theoretical) rationale for on-target variants to predict the binding affinity of targeted therapies, from the perspective of changes in structural and molecular properties of the mutated protein.^13^^,^^15^^,^^16^

### Data collection

All cases that were discussed by the UMCG-MTB between 1 January 2019 and 31 December 2020 were reviewed. Cases for which recommendations with regard to targeted therapy were made were subdivided into three categories: (i) targeted therapy recommended as the next line of treatment (targeted therapy is recommended as the first treatment to be initiated after MTB review), (ii) targeted therapy recommended after standard therapy (targeted therapy is recommended only after the patient has received available standard therapies, such as chemotherapy or immunochemotherapy, which they have not yet received at time of MTB review), or (iii) no targeted therapy recommended. For patients receiving a recommendation for targeted therapy as the next line of treatment, either on- or off-label, follow-up data were collected from electronic health records. Definitions of on- and off-label treatment were based on the possibility of prescribing the recommended targeted therapy regimen as on- or off-label treatment at the time of the MTB in 2019 and 2020 (Supplementary Table S1, available at https://doi.org/10.1016/j.esmoop.2024.103966). Exclusion criteria for follow-up were (i) requests for advice other than treatment advice for patients with histopathological diagnosis of cancer with metastatic disease, (ii) MTB-recommended targeted therapy was present in national treatment guidelines, (iii) death of the patient before MTB, (iv) incomplete documentation, and (v) lack of informed consent. Data collected included demographics, previous therapy regimens and treatment outcome, therapy regimen after MTB discussion, best overall response to treatment (according to RECIST version 1.1, without confirmation of response),^17^ date(s) of disease progression, date of stopping treatment, reason for stopping treatment, and date of death. If treatment with targeted therapy in a clinical trial was recommended, retrieved follow-up data consisted of participation status in the recommended trial (yes/no) and if applicable, the reason(s) for not participating in the trial. Results of clinical trials are published by their principal investigators and are therefore not included in this study.

The study protocol has been reviewed and approved by the OncoLifeS Scientific Board (application 202200259) and by the Central Ethics Review Board non-WMO studies of the UMCG for the inclusion of patients that have been included in the OncoLifeS Databank and the inclusion of patients who are no longer alive. For participation in the OncoLifeS Databank, patients provided written informed consent. The approval of the Central Ethics Review Board non-WMO studies of the UMCG resulted in a waiver for obtaining informed written consent for patients who are no longer alive. Patients from external hospitals were included according to the local procedure of each participating institution.

### Statistical analysis

The therapy regimen received after the MTB was compared with the MTB recommendation to determine the congruence to the MTB treatment recommendation. Progression-free survival (PFS) and overall survival (OS) were calculated by the time difference between the start of treatment and disease progression and death, respectively. In case the progressive disease was not confirmed as per RECIST version 1.1, alternative endpoints for calculation of PFS were the date of death or loss to follow-up. Cases were excluded from PFS analysis if treatment with targeted therapy was started but discontinued without confirmed progressive disease (e.g. due to toxicity) and alternative treatment was started. Study data were collected and managed using REDCap electronic data capture tools. Data analysis was carried out using SPSS version 28.0.0.0 (SPSS Inc., Chicago, IL). Swimmer plots were made using GraphPad Prism version 9.1.0 for Windows (GraphPad Software, San Diego, CA).

## Results

### UMCG-MTB targeted therapy recommendations

**Figure 1:**
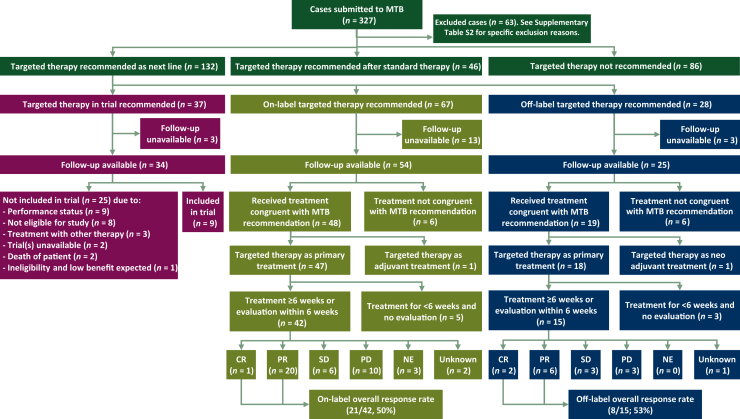
**Consolidated Standards of Reporting Trials (CONSORT) diagram of MTB recommendations, treatment setting, congruence of received treatment and recommended treatment, and response rates.** CR, complete response; MTB, molecular tumor board; NE, not evaluable; PD, progressive disease; PR, partial response; SD, stable disease.

In 2019 and 2020, a total of 327 cases were submitted for discussion by the UMCG-MTB (Figure 1), of which 265 (81%) were malignancies of pulmonary origin. Sixty-three patients were excluded because the recommended targeted therapy was in accordance with standard guidelines (*n* = 19), the advice/question was related to molecular testing (*n* = 14), discussion was not needed/no discussion in MTB (*n* = 9), or other (*n* = 21; Supplementary Table S2, available at https://doi.org/10.1016/j.esmoop.2024.103966).

Among the included 264 patients, targeted therapy was not recommended in 86 patients (33%), recommended after standard therapy in 46 patients (17%), and recommended as the next line of treatment in 132 (50%) patients. Among the latter, targeted treatment was recommended in a clinical trial setting for 37 patients, in an on-label setting for 67 patients, and in an off-label setting for 28 patients. Follow-up data were not available for 3, 13, and 3 patients, respectively.

### Targeted therapy recommended in the trial setting

Among patients for whom targeted therapy was recommended as the next line of treatment in a clinical trial setting, only 26% (9/34) were included in the recommended study (Figure 1). The most common reasons for not participating in a clinical trial were poor performance (9/25, 36%) and ineligibility due to other reasons than poor performance (8/25, 32%).

### Targeted therapy recommended in an on- or off-label setting

Follow-up data were available for 54 and 25 patients with next-line on- and off-label targeted therapy recommendations, respectively. The two most common initial (driver) gene mutations in these patients were *EGFR* mutations (47/79, 59%) and *ALK* fusions (17/79, 22%) (Supplementary Table S3, available at https://doi.org/10.1016/j.esmoop.2024.103966). Other (driver) mutations were observed in *BRAF* (*n* = 8), *MET* (*n* = 5), *ROS1* (*n* = 1), and *KIT* (*n* = 1). Molecular modeling was predominantly carried out for rare/uncommon *EGFR* mutations (*n* = 17), as well as for one *ALK* resistance mutation and one *BRAF* non-V600 mutation (Supplementary Table S3, available at https://doi.org/10.1016/j.esmoop.2024.103966). Of all patients, 85% were treated with the recommended therapy as the next line of treatment (67/79; Figure 1). Reasons for not receiving the recommended therapy were poor performance/rapid deterioration or death (*n* = 4), preference for alternative treatment option (*n* = 2), the start of other treatment before receiving the MTB advice (*n* = 3), and unknown (*n* = 3).

Of the 67 patients receiving the recommended therapy as the next line of treatment, 57 patients received their therapy as primary palliative treatment and were treated for ≥6 weeks or had a response evaluation within 6 weeks after the start of treatment. Seventy percent of these patients (40/57) had received one or more prior systemic therapies. Nearly all of these patients had received prior treatment with one or more lines of targeted therapy (37/40, 95%; Table 1). Nonpulmonary malignancies for which the recommended targeted therapy was received as the next line of treatment comprised only a small number of cases (3/57).

**Table 1:** Characteristics of patients receiving MTB-recommended targeted therapy as the next-line primary systemic treatment in the palliative setting

Characteristics	On-label (*n* = 42)	Off-label (*n* = 15)
Age (years), median (range)	69 (60-75)	65 (59-76)
Sex, *n* (%)		
Female	28 (67)	5 (33)
Male	14 (33)	10 (67)
Institution requesting MTB discussion, *n* (%)		
UMCG	24 (57)	13 (87)
External hospital	15 (36)	2 (13)
Unknown	3 (7)	0 (0)
Total number of prior lines of systemic therapy, *n* (%)		
No prior lines	14 (33)	3 (20)
One prior line	4 (10)	3 (20)
Two prior lines	13 (31)	6 (40)
Three or more prior lines	11 (26)	3 (20)
Prior chemotherapy, *n* (%)		
One prior line	10 (24)	5 (33)
Two prior lines	3 (7)	1 (7)
Prior chemoimmunotherapy, *n* (%)	5 (12)	0 (0)
Prior monoimmunotherapy, *n* (%)	2 (5)	2 (13)
Prior targeted therapy, *n* (%)		
One prior line	8 (19)	5 (33)
Two prior lines	11 (26)	4 (27)
Three prior lines	4 (10)	2 (13)
Four or more prior lines	4 (10)	0 (0)
Tumor type, *n* (%)		
Lung adenocarcinoma	39 (93)	14 (93)
Melanoma	2 (5)	1 (7)
Lung squamous cell carcinoma	1 (2)	0 (0)

MTB, molecular tumor board; UMCG, University Medical Center Groningen.

### Treatment outcome—on-label targeted therapy

Five patients did not receive targeted therapy treatment for at least 6 weeks nor were evaluated with imaging at an earlier timepoint and were therefore not included in the overall response rate evaluation (Figure 1). Patients receiving the recommended targeted therapy as a primary systemic treatment in an on-label setting had an overall response rate of 50% (21/42). One patient with metastatic melanoma had an unknown response due to the unavailability of measurable lesions, though a clinical response was described. The median PFS was 6.3 months [interquartile range (IQR) 2.9-14.9 months] and the median OS was 15.8 months (IQR 6.4-34.2 months); 11 (26%) patients were alive at the latest follow-up.

**Figure 2:**
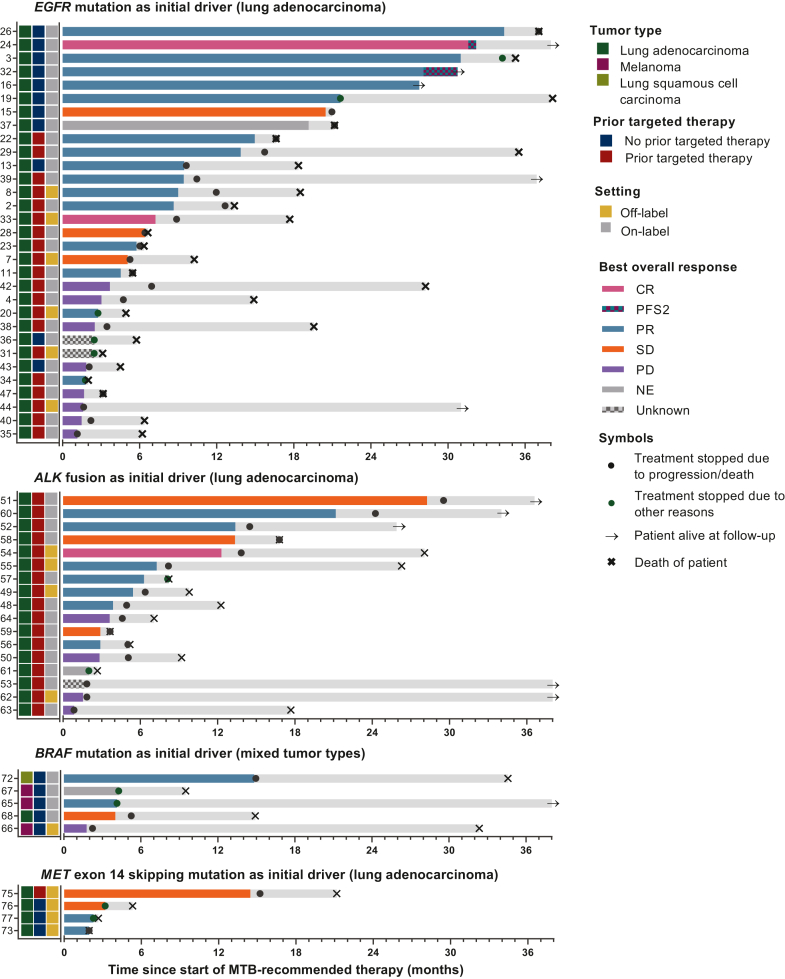
**Swimmer plots of patients receiving targeted therapy as recommended by the UMCG-MTB grouped per initial driver mutation.** ALK, anaplastic lymphoma kinase; CR, complete response; EGFR, epidermal growth factor receptor; UMCG-MTB, Molecular Tumor Board of the University Medical Center Groningen; NE, not evaluable; PD, progressive disease; PFS2, second progression-free survival (if the patient received local treatment for locoregional progression or oligometastatic disease and continued systemic treatment); PR, partial response; SD, stable disease. For not evaluable, unknown responses, and initial responses without progression date (e.g. due to a switch of therapy), the bars depict the duration of treatment instead of progression-free survival. Light gray bars depict the overall follow-up time. For patient #15, the best supportive care was initiated after disease progression and the patient died (date of death unknown).

A swimmer plot was created to visualize PFS and OS for all patients (Figure 2). Patients with uncommon *EGFR*-mutated lung adenocarcinoma for whom treatment guidelines in 2019 and 2020 were lacking and who were treated with on-label targeted therapy demonstrated relatively high response rates and long PFS. Moreover, patients treated with on-label therapy had longer PFS compared with patients treated with off-label therapy.

Reasons to stop treatment were progression of disease (23/42, 55%), toxicity (5/42, 12%), complications of disease/other disease (2/42, 5%), and therapy switch not due to progression of disease or toxicity (2/42, 5%). Seven patients continued treatment until or stopped treatment shortly before their death, and three patients were still treated with the recommended therapy at the latest follow-up.

An overview of MTB recommendations for patients with progressive disease at initial response evaluation is depicted in Table 2. An overview of all MTB recommendations with targeted therapy recommended as the next line of treatment is available in Supplementary Table S3, available at https://doi.org/10.1016/j.esmoop.2024.103966.

**Table 2:** Patients receiving MTB-recommended treatment and who had progressive disease at initial response evaluation (on-label recommendations only)

ID	Previous treatment lines^a^	Aberrations	MTB recommendation	Treatment	BOR	PFS (months)
***EGFR* mutation as the primary driver (all pulmonary malignancies)**						
42	1. Afatinib [*EGFR* p.(L858R)] (stop due to toxicity) 2. Gefitinib	*EGFR* p.(L858R) *EGFR* p.(T790M) (very low allelic frequency) *TP53* p.(R156C) (very low allelic frequency)	Osimertinib	Osimertinib	PD	3.7
4	1. Gefitinib [*EGFR* p.(L858R)] 2. Immunotherapy 3. Gefitinib + chemotherapy 4. Osimertinib [*EGFR* p.(L858R), *EGFR* p.(T790M)] 5. Docetaxel + nintedanib [*EGFR* p.(L858R), *EGFR* p.(T790M), *EGFR* p.(C797S)] (stop due to toxicity)	*EGFR* p.(L858R) *EGFR* p.(T790M) Loss of previously detected *EGFR* p.(C797S)	Rechallenge osimertinib	Osimertinib	PD	3.0
38	1. Gefitinib [*EGFR* p.(L747_T751del)] 2. Erlotinib 3. Chemotherapy 4. Osimertinib [*EGFR* p.(L747_T751del), *EGFR* p.(T790M)]	*EGFR* p.(L747_T751del) Loss of previously detected *EGFR* p.(T790M)	Rechallenge erlotinib	Erlotinib	PD	2.5
35	1. Afatinib [*EGFR* p.(L747_A750insP)] 2. Osimertinib [*EGFR* p.(L747_A750delinsP), *EGFR* p.(T790M)]	*EGFR* p.(L747_A750delinsP) *BRAF* p.(V600E) Loss of previously detected *EGFR* p.(T790M)	Osimertinib + dabrafenib + trametinib	Dabrafenib + trametinib started	PD	1.1
43	1. Gefitinib [*EGFR* p.(T751_I759delsinsN)] 2. Osimertinib [*EGFR* p.(T751_I759delsinsN), *EGFR* p.(T790M)]	*EGFR* p.(T751_I759delinsN) *EGFR* p.(G724S) Loss of *EGFR* T790M Potential *RET* fusion (NanoString +, FISH RET –)	Osimertinib + afatinib or afatinib monotherapy	Afatinib (reduced dose due to toxicity)	PD	1.8
47	1. Chemotherapy (stop due to molecular test results) 2. Gefitinib (*EGFR* p.(E746_A750del) and *RET* fusion based on FISH)	*EGFR* p.(E746_A750del) *EGFR* p.(T790M) *KRAS* p.(G13D) Loss of previously detected *RET* fusion	Osimertinib	Osimertinib	PD	1.7
40	1. Gefitinib [*EGFR* p.(E746_A750del)] 2. Osimertinib [*EGFR* p.(E746_A750del), *EGFR* p.(T790M)] 3. Osimertinib + crizotinib [*EGFR* p.(E746_A750del), *MET* amplification, loss of *EGFR* p.(T790M)]	*EGFR* p.(E746_A750del) *BRAF* p.(V600E) Loss of *MET* amplification Persistent loss of *EGFR* T790M	Osimertinib + dabrafenib + trametinib	Osimertinib + dabrafenib + trametinib	PD	1.5
***ALK* fusion as the primary driver (all pulmonary malignancies)**						
62	1. Alectinib	ALK IHC+ No resistance mechanism detected	Higher dose alectinib	Alectinib	PD	1.5
15	1. Crizotinib 2. Alectinib	*ALK* fusion transcript, NOS *ALK* p.(L1196Q)	Ceritinib	Ceritinib	PD	2.6
64	1. Chemotherapy 2. Crizotinib (*ALK* fusion) 3. Ceritinib 4. Alectinib 5. Lorlatinib 6. Chemoimmunotherapy	*EML* exon 13::*ALK* exon 20 fusion No *ALK*-resistance mutations found	Rechallenge previous ALK-TKI	Alectinib	PD	3.6

ALK, anaplastic lymphoma kinase; BOR, best overall response; EGFR, epidermal growth factor receptor; IHC, immunohistochemistry; MTB, molecular tumor board; NOS, not otherwise specified; PD, progressive disease; PFS, progression-free survival; TKI, tyrosine kinase inhibitor.

a Previous treatment lines are displayed in chronological sequence. Unless denoted otherwise, previous lines of treatment were stopped due to disease progression. New molecular testing results before treatment are noted in parentheses. Standard treatment was preferred by MTB.

### Treatment outcome—off-label targeted therapy

Three patients did not receive targeted therapy treatment for at least 6 weeks nor were evaluated with imaging at an earlier timepoint and were therefore not included in the overall response rate evaluation. Patients receiving recommended targeted therapy as primary systemic treatment in an off-label setting had an overall response rate of 53% (8/15; Table 3 and Figure 1). The median PFS was 5.1 months (IQR 1.9-7.3 months) and the median OS was 17.7 months (IQR 5.1-23.7 months). One patient was alive at the latest follow-up.

**Table 3:** Patients who received MTB-recommended off-label treatment with targeted therapy

ID	Previous treatment lines^a^	Aberrations	MTB recommendation	Treatment	BOR	PFS (months)
***EGFR* mutation as the primary driver (all pulmonary malignancies)**						
33	1. Gefitinib [*EGFR* p.(E746_A750del)] 2. Osimertinib [*EGFR* p.(E746_A750del), *EGFR* p.(T790M)]	*EGFR* p.(E746_A750del) *MET* c.2942-30_2952del (*MET* exon 14 skipping)	Osimertinib + crizotinib	Osimertinib + crizotinib	CR	7.2
8	1. Gefitinib [*EGFR* p.(E746_A750del)] 2. Osimertinib [*EGFR* exon 19 deletion, *EGFR* p.(T790M)]	*EGFR* p.(E746_A750del) *MET* amplification Loss of *EGFR* p.(T790M)	Osimertinib + crizotinib	Osimertinib + crizotinib	PR	9.0
20	1. Chemotherapy (started awaiting molecular test results) 2. Afatinib [*EGFR* p.(E746_A750del)]	*EGFR* p.(E746_A750del) *MET* amplification *ERBB2* polysomy	Afatinib + crizotinib	Afatinib + crizotinib	PR	n/a
7	1. Gefitinib [*EGFR* p.(E746_A750del)] 2. Osimertinib [*EGFR* p.(E746_A750del), *EGFR* p.(T790M), *PTEN* p.(D92H)] 3. Chemotherapy 4. Osimertinib (started awaiting molecular test results)	*EGFR* p.(E746_A750del) *PTEN* deletion No *EGFR* p.(T790M) No *PIK3CA* mutation	Osimertinib + everolimus	Osimertinib + everolimus	SD	5.1
44	1. Osimertinib [*EGFR* exon 19 deletion mutation and *EGFR* p.(T790M) (primary resistance mutation)]	*EGFR* p.(L747_P753delinsS) *EGFR* p.(T790M) *EGFR* p.(C797S)	Brigatinib	Brigatinib	PD	1.6
14	1. Afatinib [*EGFR* p.(L858R), stopped due to toxicity] 2. Gefitinib	*EGFR* p.(L858R) *KRAS* p.(T50I) *MET* amplification (low)	Crizotinib + EGFR TKI	Started with crizotinib, to be followed by EGFR TKI	NE	n/a (death of the patient before the first evaluation)
17	1. Gefitinib (*EGFR* exon 19 deletion) 2. Osimertinib [*EGFR* p.(E746_A750del), *EGFR* p.(T790M)]	*EGFR* p.(E746_A750del) *EGFR* p.(T790M) *MET* amplification	Osimertinib + crizotinib	Osimertinib + crizotinib	NE	n/a (death of the patient before the first evaluation)
31	1. Chemotherapy 2. Gefitinib [*EGFR* p.(E746_A750del)]	*EGFR* p.(E746_A750del) *MET* amplification	Gefitinib + crizotinib	Gefitinib + crizotinib	NE	3.1 (death of the patient, treatment stopped 2 weeks prior because of toxicity)
***ALK* fusion as the primary driver (all pulmonary malignancies)**						
54	1. Crizotinib (*ALK* fusion) 2. Ceritinib [*ALK* fusion, *ALK* p.(L1196Q)]	*EML4* exon 2::*ALK* exon 20 fusion *ALK* p.(F1174V) Loss of *ALK* p.(L1196Q)	Lorlatinib (not yet on-label at the date of MTB)	Lorlatinib	CR	12.3
55	1. Alectinib (*ALK* fusion)	*EML4* exon 6::*ALK* exon 20 fusion *ALK* p.(V1180L)	Ceritinib	Ceritinib	PR	7.3
49	1. Chemotherapy 2. Alectinib 3. Ceritinib (started awaiting molecular test results, stopped due to toxicity) 4. Lorlatinib (started awaiting molecular test results, stopped due to toxicity)	*EML4* exon 2::*ALK* exon 20 fusion *MET* amplification	Crizotinib	Crizotinib	PR	5.4
63	1. Alectinib (*EML4*_6a::*ALK*_20)	*EML4*_6a::*ALK*_20 (previously detected) Additional molecular testing was not possible	Ceritinib	Ceritinib	PD	2.5
***MET* exon 14 skipping mutation as the primary driver (all pulmonary malignancies)**						
77	None	*MET* c.3072_3082+4del (*MET* exon 14 skipping)	Crizotinib	Crizotinib	PR	2.7 (death of the patient, treatment stopped 2 weeks prior due to toxicity and clinical suspicion for progression)
73	None	*MET* c.2913_2914delinsT (*MET* exon 14 skipping) *PIK3CA* p.(H1047R) *IDH2* p.(R140Q)	Crizotinib	Crizotinib	PR	1.9 (death of the patient)
75	1. Chemotherapy 2. Chemotherapy 3. Therapy in trial 4. Crizotinib	*MET* exon 14 skipping	Capmatinib	Capmatinib	SD	14.5
76	1. Chemotherapy 2. Immunotherapy	*MET* c.3082G>C (*MET* exon 14 skipping)	Crizotinib	Crizotinib	SD	n/a
74	None	*MET* c.3028G>C (*MET* exon 14 skipping)	Crizotinib	Crizotinib	NE	n/a (death of the patient before first evaluation)
***BRAF* mutation as primary driver (melanoma)**						
66	None	*BRAF* p.(T599dup)	BRAFi + MEKi possible^b^	Dabrafenib + trametinib	PD	1.8

ALK, anaplastic lymphoma kinase; BOR, best overall response; BRAFi, BRAF inhibitor; CR, complete response; EGFR, epidermal growth factor receptor; MEKi, MEK inhibitor; MTB, molecular tumor board; NE, not evaluable; PD, progressive disease; PFS, progression-free survival; PR, partial response; SD, stable disease; TKI, tyrosine kinase inhibitor.

a Previous treatment lines are displayed in chronological sequence. Unless denoted otherwise, previous lines of treatment were stopped due to disease progression. New molecular testing results before treatment are noted in parentheses.

b With warning of potentially reduced efficacy. Standard treatment was preferred by the MTB.

Reasons to stop treatment after initial response evaluation were a progression of disease (10/15, 67%), toxicity (3/15, 20%; for 1 patient progression was also suspected clinically), and switch to a different therapy not due to disease progression or toxicity (1/15, 7%). The remaining patients continued treatment until or stopped treatment shortly before their death.

### Treatment outcome—3D modeling

Patients for whom modeling was carried out and recommended targeted therapy was prescribed (as primary treatment and received for at least six weeks) had an overall response rate of 54% (7× partial response, 3× progressive disease, 3× not evaluable, Supplementary Table S3, available at https://doi.org/10.1016/j.esmoop.2024.103966).

## Discussion

In this study, we describe the characteristics and outcomes of patients with cancer with uncommon or rare (combinations of) mutational profiles who received next-line targeted therapy as recommended by the UMCG-MTB in 2019 and 2020. Most of these patients had received at least two prior systemic therapies (58%). Treatment with MTB-recommended next-line on- or off-label targeted therapy resulted in a partial or complete response in 50% and 53% of patients, respectively.

Half of the MTB treatment recommendations comprised targeted therapies as the next line of treatment, as either on- or off-label therapy, while the remaining recommendations consisted of targeted therapy after standard therapy (∼1/3) or targeted therapy not advised (∼2/3). Recommended targeted therapy as the next-line treatment, either on- or off-label, was received by 85% (67/79) of patients, which is comparable to that of the UMCG-MTB follow-up study of patients discussed in 2018.^13^ Direct comparison of response rates with the previous follow-up study is not possible, as the definition of on- and off-label prescription of targeted therapies used in this study differs from the previous study. In the previous study, treatment was considered on-label if the treatment was ‘labeled for the specific molecular indication or described in the guidelines at the time of recommendation and off-label if these criteria were not met’.^13^ In contrast, in this study, patients were excluded when the recommended therapy was described in national guidelines (*n* = 19), to prevent inclusion of noncomplex cases. Instead, definitions of on- and off-label treatment were based on the possibility of prescribing the recommended targeted therapy regimen as on- or off-label treatment at the time of the MTB. In general, (international) comparison of MTB functioning and treatment outcome is complicated, mainly due to differences in the patient populations that are discussed in MTBs, but also due to differences in molecular tests (e.g. gene content of the panels), application of testing (e.g. testing at disease progression), treatment guidelines, and the availability of (targeted) therapies.^6^

Treatment with MTB-recommended off-label targeted therapy did not negatively affect the response rate to treatment (53% compared with 50% in patients treated on-label). Of note, MET-targeted therapy [either alone (*n* = 5) or in combination with other agents (*n* = 2)] was the most commonly used off-label targeted therapy in patients with either primary *MET* aberrations or acquired *MET* aberrations after epidermal growth factor receptor (EGFR)- or anaplastic lymphoma kinase (ALK)-targeted therapy. By now, the European Medicines Agency (EMA) has authorized the use of capmatinib and tepotinib in patients with advanced-stage NSCLC harboring a *MET* exon 14 skipping mutation after prior treatment with immunotherapy and/or platinum-based chemotherapy.^18^^,^^19^ Crizotinib, by contrast, has been EMA approved only for patients with *ALK* fusion-positive or *ROS1* fusion-positive NSCLC.^20^ Moreover, none of these therapies have been approved by the EMA for tumors with an amplification of *MET*. In this study, we described a series of patients with NSCLC with an acquired *MET* aberration after EGFR tyrosine kinase inhibitor (TKI) treatment and we show that crizotinib may be an effective additional therapeutic option (in addition to EGFR TKI) for the subsequent treatment of these patients. A Danish study described a similar effective treatment outcome in five patients with concurrent *EGFR*-mutated and *MET*-amplified metastatic NSCLC who were treated with combined EGFR TKI and crizotinib.^21^ Another recent case series described the benefit of combined tepotinib and EGFR TKI treatment in patients with MET-amplified NSCLC upon progression on a previous EGFR TKI.^22^ Our results demonstrate the effectiveness of MTB-recommended off-label prescription of specific targeted therapies. When a rationale for off-label use is provided by an MTB, getting access to these drugs for treatment (e.g. in compassionate use) is often challenging. Based on our results, easier access to off-label use of targeted drugs is warranted as it may facilitate more optimal treatment outcomes for patients with specific mutational profiles. The increase in the number of patients treated with off-label targeted therapy regimens will provide more robust clinical data and thereby be beneficial for treatment advice for future patients.

Progressive disease at initial response evaluation was observed in 24% (10/42) of patients treated with on-label targeted therapy. Five out of these ten patients, each with an *EGFR* mutation as an initial driver, had a loss of a previously acquired resistance mechanism, including *EGFR* p.(C797S) and p.(T790M) mutations. For three of these five patients, rechallenge with a previously prescribed targeted therapy or a targeted therapy previously considered ineffective due to the molecular profile was initiated. Limited effectiveness has been described in patients treated with rechallenge of EGFR TKI after prior treatment with EGFR TKI (first line) and chemotherapy (second line).^23^^,^^24^ Importantly, the definition of ‘rechallenge’ is not uniform throughout the literature, as some studies use the term to refer to any EGFR TKI after any prior EGFR TKI rather than rechallenge with the same specific TKI.^23^^,^^24^

The remaining two patients with loss of previously acquired resistance mechanisms both had a *BRAF* p.(V600E) mutation as a newly acquired resistance mechanism. Combination treatment with BRAF/MEK inhibitors and osimertinib was recommended for either patient, but both experienced disease progression at the next evaluation after treatment initiation. For patients with acquired *BRAF* p.(V600E) mutation after EGFR TKI treatment, combined BRAF/MEK inhibitor and EGFR TKI has previously been described as an effective treatment strategy, although this has been limited to case reports.^25^^,^^26^ However, in one case report, the patient did not lose a previously acquired resistance mechanism. The other case report was of the patient who was also included in this study.^26^

Among the other five patients with progressive disease at initial response evaluation after on-label therapy, there were three patients with an *ALK* fusion as an initial driver. Two patients had no detected on- or off-target resistance mutations after ALK inhibitor treatment. One patient was treated with ceritinib upon an acquired *ALK* p.(L1196Q) mutation after prior treatment with alectinib and crizotinib. This rare *ALK* mutation has later been described as an acquired resistance mechanism to ALK-targeted therapy with low binding affinity for both ceritinib and lorlatinib based on computational modeling.^27^ The third-generation ALK inhibitor lorlatinib was only approved by the EMA at the end of February 2019, and for some patients with resistance to ALK-targeted therapy that were discussed in the MTB, current-day recommendations would likely include lorlatinib as the preferred next line of treatment.

The remaining two patients with progressive disease at initial response evaluation had both been treated with EGFR TKI. One patient had an *EGFR* p.(T790M) resistance mutation with low-variant allele frequency in comparison to the *EGFR* driver mutation and the other patient with an *EGFR* p.(E746_A750del) had acquired a concurrent *EGFR* p.(T790M) and *KRAS* mutation after prior EGFR TKI. Both patients were recommended to receive osimertinib as the next line of treatment and had progressive disease at first treatment evaluation, potentially due to other nondetected resistance mechanism(s) or the detected *KRAS* mutation reducing the efficacy of osimertinib, respectively.

*In silico* mutational modeling and docking results have been applied as a tool to predict potential utility in predicting drug responses in *EGFR*-mutant NSCLC,^28^^,^^29^ *BRAF*-mutant NSCLC,^30^ *BRAF*-mutant melanoma,^31^ and *ALK*-mutated NSCLC.^32^ The UMCG-MTB carries out molecular modeling for patients with uncommon and rare (combinations of) mutations, if insufficient treatment data are available in the literature. In our cohort, patients for whom modeling was carried out and recommended targeted therapy was prescribed (as primary treatment and for at least 6 weeks) had an overall response rate of 54%, which is similar to the response rate of modeling cases of the UMCG-MTB in 2018 (50%).^13^ Nevertheless, the actual value of modeling for treatment decision making in patients with cancer with uncommon rare (combinations of) mutational profiles still requires experimental validation.

Analyzing MTB follow-up data requires an understanding of the situation at the time of the MTB recommendation. It is important to realize that these recommendations can become common practice only a few years later. At the time of case discussion, MTBs function as pioneers in the field of targeted treatment and have the opportunity to create a path or leave a trail to optimize treatment for future patients. With periodic evaluation, MTBs can collect positive reinforcing information (e.g. use of off-label therapies in this cohort) as well as information indicating reconsideration of a (specific) targeted therapy as an optimal treatment option in future patients with comparable characteristics (e.g. loss of previously detected resistance mechanisms).

As stated earlier, comparing the effectiveness of different MTBs is fraught with difficulties, including the specific aims of an MTB (e.g. routine diagnostics, research diagnostics, specific tumor types), case selection (e.g. all patients or limited to rare molecular profiles), composition and workflow, and molecular tests and reporting approaches (e.g. single-gene testing, broad-panel NGS). Additional complicating factors are the availability of on-label targeted therapies and their reimbursement, as well as the availability of compassionate use programs and clinical trials that differs among various countries. A previous suggestion to describe effectiveness of MTBs includes the use of the PFS2-to-PFS1 ratio or ‘von Hoff ratio’ (i.e. the ratio of PFS of prior systemic treatment to PFS of MTB-recommended treatment) with a fixed cut-off value between 1.3 and 1.5.^33^^,^^34^ However, this approach has its own limitations (e.g. unusual efficacy of prior treatment, variation in number of prior lines) and does not correct for other issues, such as case selection and treatment availability.^35^ A modified version of this ratio has been developed to more accurately resemble therapeutic benefits as evaluated by clinicians.^35^ Nevertheless, the relevance and applicability of these ratios to the assessment of MTB outcome is unclear. In our opinion, periodic retrospective MTB follow-up studies are predominantly useful for (i) the expansion of real-world evidence of specific targeted therapies as treatment in patients with rare molecular aberrations (relevant to future patients), (ii) self-evaluation of MTB functioning (steps for optimization), and (iii) trend analysis with regard to patient characteristics, mutational profile types, and use of specific (combinations of) targeted therapies (e.g. changes in patient populations, unmet needs). As not all real-world data of MTBs are currently collected and/or available to healthcare professionals, regional or (inter)national collaborations to harmonize periodic review of MTB-recommended treatment outcomes and make these data available in a centralized database accessible for other MTBs will be useful.

Patients with lung cancer comprised the vast majority of the patient population of our study. However, over the last few years, options for targeted therapy in other malignancies have become increasingly available. This is reflected by the change in the composition of the patient population reviewed by the UMCG-MTB. While patients with pulmonary malignancies comprised >80% of cases reviewed by the UMCG-MTB in 2019/2020, the distribution is approximately half pulmonary and nonpulmonary malignancies in 2023/2024. Depending on changes in guidelines and the availability of targeted therapies, approved or in clinical trials, it is expected that the composition of the reviewed patient population will continue to shift.

In conclusion, most patients in this retrospective follow-up cohort received MTB-recommended next-line targeted therapy. These treatments resulted in positive overall responses in over half of, often heavily pretreated, patients with uncommon or rare (combinations of) mutations for which current guidelines provide insufficient guidance. Off-label use of targeted therapies, for which there was sufficient rationale as determined by our MTB, was also an effective treatment strategy. Lowering the threshold of access to off-label targeted therapies may facilitate more optimal treatment of patients with specific molecular tumor profiles. This study underscores the relevance of discussing patients with rare or uncommon mutations in an MTB.
