# Supplementary material for: Molecular Tumor Board of the University Medical Center Groningen (UMCG-MTB): outcome of patients with rare or complex mutational profiles receiving MTB-advised targeted therapy
Source: ESMO Open. 2024 Nov 4;9(11):103966. doi: 10.1016/j.esmoop.2024.103966 (PMC11570463; doi:10.1016/j.esmoop.2024.103966)
Supplement: Supplementary Table S1 [file mmc1.docx]

| **Supplementary Table S1.** Overview of on-label/off-label setting of targeted therapies in 2019-2020 | |
| --- | --- |
| Afatinib | Considered on-label for any *EGFR* mutation(s) if prescribed as first line of treatment. |
| Afatinib + crizotinib | Considered on-label for combination of any *EGFR* mutation(s) and *ALK* fusion. Considered off-label for combination of any *EGFR* mutation(s) and *MET* exon 14 skipping mutation or *MET* amplification. |
| Alectinib | Considered on-label for any *ALK* fusion. |
| Capmatinib | Considered off-label. |
| Ceritinib | Considered on-label for any *ALK* fusion if prescribed as first line of treatment or after prior crizotinib treatment. |
| Crizotinib | Considered on-label for any *ALK* fusion.  Considered off-label for *MET* exon 14 skipping mutation or *MET* amplification. |
| Dabrafenib + trametinib | Considered on-label for any *BRAF* V600 mutation. |
| Erlotinib | Considered on-label for any *EGFR* mutation(s). |
| Gefitinib | Considered on-label for any *EGFR* mutation(s). |
| Gefitinib + crizotinib | Considered on-label for combination of any *EGFR* mutation(s) and *ALK* fusion.  Considered off-label for combination of any *EGFR* mutation(s) and *MET* exon 14 skipping mutation or *MET* amplification. |
| Lorlatinib | From 28 February 2019, considered on-label after alectinib or ceritinib as first line of treatment, or after treatment with crizotinib and one other ALK inhibitor. |
| Osimertinib | Considered on-label for any *EGFR* mutation(s) if prescribed as first line of treatment or in the presence of *EGFR* T790M mutation. |
| Osimertinib + alectinib | Considered on-label for combination of any *EGFR* mutation(s) and *ALK* fusion. |
| Osimertinib + crizotinib | Considered on-label for combination of any *EGFR* mutation(s) and *ALK* fusion. Considered off-label for combination of any *EGFR* mutation(s) and *MET* exon 14 skipping mutation or *MET* amplification. |
| Osimertinib + dabrafenib + trametinib | Considered on-label for combination of any *EGFR* mutation(s) and *BRAF* V600 mutation. |
| Osimertinib + erlotinib | Considered on-label for any *EGFR* mutation(s). |
| Osimertinib + everolimus | Considered off-label. |
| Brigatinib | Considered on-label for any *ALK* fusion if prescribed after prior treatment with crizotinib.  Considered off-label for any *EGFR* mutation(s). |
