# Supplementary material for: Molecular Tumor Board of the University Medical Center Groningen (UMCG-MTB): outcome of patients with rare or complex mutational profiles receiving MTB-advised targeted therapy
Source: ESMO Open. 2024 Nov 4;9(11):103966. doi: 10.1016/j.esmoop.2024.103966 (PMC11570463; doi:10.1016/j.esmoop.2024.103966)
Supplement: Supplementary Table S2 [file mmc2.docx]

| **Supplementary Table S2.** Specified reasons for study exclusion | |
| --- | --- |
| Reason for exclusion | Number of cases |
| Targeted therapy is standard treatment/recommendation present in treatment guidelines | 19 |
| Advice/question related to molecular diagnostics | 14 |
| Case not suited for MTB/discussion in MTB not needed/not discussed in MTB | 9 |
| Additional molecular testing required | 4 |
| Incomplete documentation | 4 |
| Patient died prior to MTB | 3 |
| Follow-up/educational case | 3 |
| No treatment referral possible due to COVID-19 pandemic | 2 |
| No informed consent form | 2 |
| Advice/question related to application of targeted therapy in early-stage disease | 1 |
| Treatment recommendation due to toxicity on other targeted therapy | 1 |
| Uncertain tumor origin (potentially more than one primary tumor) | 1 |
| Total number of excluded cases | 63 |
