# Supplementary material for: Molecular Tumor Board of the University Medical Center Groningen (UMCG-MTB): outcome of patients with rare or complex mutational profiles receiving MTB-advised targeted therapy
Source: ESMO Open. 2024 Nov 4;9(11):103966. doi: 10.1016/j.esmoop.2024.103966 (PMC11570463; doi:10.1016/j.esmoop.2024.103966)
Supplement: Supplementary Table S3 [file mmc3.docx]

| **Supplementary Table S3**. Treatment recommendations and outcomes of patients with available follow-up information. | | | | | | | | | | | |
| --- | --- | --- | --- | --- | --- | --- | --- | --- | --- | --- | --- |
| **ID** | **L** | **Aberrations** | **Recommendation of UMCG-MTB** | **Modeling** | **Congruence** | **Treatment** | **Setting** | **BOR** | **PFS** | **DoT** | **OS** |
| ***EGFR* mutation as (initial) driver mutation** | | | | | | | | | | | |
| 1 | 0 | *EGFR* p.(L703F) | Afatinib (though limited evidence it concerns activating) | Yes | Yes | Afatinib | On-label | NE | 1.0 | 1.0 | 1.0 |
| 2 | 2 | *EGFR* p.(L747_P753delinsS)  *EML4* exon 2::*ALK* exon 20 fusion | Osimertinib + alectinib | No | Yes | Osimertinib +  alectinib | On-label | PR | 8.6 | 12.6 | 13.4 |
| 3 | 0 | *EGFR* p.(L858R)  *EGFR* amplification | EGFR TKI. If rapid progression, consider adding cetuximab. | No | Yes | Osimertinib | On-label | PR | 31.0 | 34.2 | 35.2 |
| 4 | 5 | *EGFR* p.(L858R)  *EGFR* p.(T790M)  Loss of *EGFR* p.(C797S) | Rechallenge osimertinib | No | Yes | Osimertinib | On-label | PD | 3.0 | 4.7 | 14.9 |
| 5 | 1 | *EGFR* p.(G719A)  *EGFR* p.(G719C)  *TP53* p.(L130P) | EGFR TKI | Yes | Yes, but treated <6 weeks | Osimertinib | On-label | NE | Unknown | Unknown | Unknown |
| 6 | 1 | *EGFR* p.(G719S) | Afatinib (afatinib preferred to osimertinib) | Yes | No (osimertinib already started) | Osimertinib | On-label |  |  |  |  |
| 7 | 4 | *EGFR* p.(E746_A750del)  *PTEN* deletion  No *EGFR* p.(T790M)  No *PIK3CA* mutation | EGFR TKI + everolimus | No | Yes | Osimertinib + everolimus | Off-label | SD | 5.1 | 5.3 | 10.2 |
| 8 | 2 | *EGFR* p.(E746_A750del)  *MET* amplification  Loss of *EGFR* p.(T790M) | Osimertinib + crizotinib | No | Yes | Osimertinib +  crizotinib | Off-label | PR | 9.0 | 12.0 | 18.5 |
| 9 | 0 | *EGFR* p.(L858R)  *EGFR* p.(K757M) | EGFR TKI based on L858R mutation | Yes | Yes, but treated <6 weeks | Afatinib | On-label | NE | n/a | 0.5 (toxicity) | 3.1 |
| 10 | 0 | *EGFR* p.(S768I)  *EGFR* p.(V774M)  *PIK3CA* p.(E545K) | Osimertinib | Yes | No (reason unknown) | Afatinib | On-label | PD | 1.5 | 1.5 | 4.4 |
| 11 | 5 | *EGFR* p.(G719A)  *EGFR* p.(T790M)  *EGFR* p.(R776G)  *EGFR* amplification (low, based on NGS) | Osimertinib | Yes | Yes | Osimertinib | On-label | PR | 4.5 | 5.5 | 5.5 |
| 12 | 0 | *EGFR* amplification | If EGFR amplification confirmed in new biopsy, then consider treatment with necitumumab | No | No (best supportive care preferred) | Best Supportive Care | Off-label |  |  |  |  |
| 13 | 0 | *EGFR* p.(I740_K745dup) | Afatinib | No | Yes | Afatinib | On-label | PR | 9.5 | 9.6 | 18.4 |
| 14 | 2 | *EGFR* p.(L858R)  *KRAS* p.(T50I)  *MET* amplification (low) | Crizotinib + EGFR TKI (start crizotinib, followed by re-introduction of EGFR TKI) | No | Yes, but treated <6 weeks | Crizotinib started | Off-label | NE | 0.8 | 0.8 | 0.8 |
| 15 | 0 | *EGFR* p.(K739_I744dup)  *CTNNB1* p.(Ser37Phe) (note: not known during MTB discussion) | Osimertinib (note: recommendation was affected by treatment accessibility due to COVID pandemic) | No | Yes | Osimertinib | On-label | SD | 20.5 | 21.0 | Unknown, best supportive care after stop osimertinib |
| 16 | 0 | *EGFR* p.(L747_E749)  *EGFR* p.(K754E)  *PIK3CA* p.(G1049R)  *MAP2K1* amplification | Osimertinib | Yes | Yes | Osimertinib | On-label | PR | n/a | 27.8 (ongoing) | 27.8  (alive) |
| 17 | 2 | *EGFR* p.(E746_A750del)  *EGFR* p.(T790M)  *MET* amplification | Osimertinib + crizotinib | No | Yes, but treated <6 weeks | Osimertinib +  crizotinib | Off-label | NE | <0.1  (death) | <0.1  (death) | <0.1 |
| 18 | 0 | *EGFR* p.(L747_T751del)  *EGFR* p.(S768I)  *TP53* p.(R213*) | Afatinib or gefitinib | Yes | No (osimertinib preferred due to toxicity profile) | Osimertinib | On-label |  |  |  |  |
| 19 | 0 | *EGFR* p.(G719S)  *EGFR* p.(L861Q) | Afatinib | Yes | Yes | Afatinib | On-label | PR | n/a | 21.6  (switch to osimertinib) | 40.2 |
| 20 | 2 | *EGFR* p.(E746_A750del)  *MET* amplification  *ERBB2* polysomy | EGFR-TKI + crizotinib | No | Yes | Afatinib +  crizotinib | Off-label | PR | n/a | 2.8 (toxicity) | 4.9 |
| 21 | 1 | *EGFR* p.(L858R)  *EGFR* p.(G598V) | Chemotherapy or afatinib | No | No (osimertinib already started) | Osimertinib | On-label | NE |  |  |  |
| 22 | 2 | *EGFR* p.(L747_T751delinsP)  *EML4* exon 2::ALK exon 20 fusion  *EGFR* amplification | Osimertinib + alectinib | No | Yes | Osimertinib +  alectinib | On-label | PR | 15.0 | 16.6 | 16.6 |
| 23 | 1 | *EGFR* p.(L858R)  *EGFR* p.(T790M) | Osimertinib | No | Yes | Osimertinib | On-label | PR | 5.7 | 6.0 | 6.3 |
| 24 | 0 | *EGFR* p.(L858R)  *PIK3CA* p.(E545K) | Osimertinib | No | Yes | Osimertinib | On-label | CR | 31.6 (PFS1)  32.2 (PFS2) | 42.8 (ongoing) | 42.8  (alive) |
| 25 | 1 | *EGFR* p.(G719C)  *EGFR* p.(S768I)  *MDM2* amplification (high; based on NGS)  *EGFR* amplification (low; based on NGS) | Add cetuximab to EGFR TKI treatment (alternative: chemo-immunotherapy) | No | No |  | Off-label |  |  |  |  |
| 26 | 0 | *EGFR* p.(G719C)  *EGFR* p.(S768I) | EGFR TKI | Yes | Yes | Gefitinib | On-label | PR | 34.4 | 37.1 | 37.1 |
| 27 | 1 | *EGFR* p.(L858R)  *MET* amplification (high)  HER pos | Add crizotinib to current treatment in case of extracerebral progression | No | No (poor performance) |  | Off-label |  |  |  |  |
| 28 | 6 | *EGFR* p.(L747_T751del)  Loss of *EGFR* p.(T790M) (still) | Rechallenge osimertinib | No | Yes | Osimertinib | On-label | SD | 6.4 | 6.4 | 6.6 |
| 29 | 2 | *EGFR* p.(G719C)  *EGFR* p.(S768I)  *EGFR* p.(T790M)  *EGFR* p.(C797S) (in trans with *EGFR* p.(T790M)) | Osimertinib + erlotinib | No | Yes | Osimertinib +  erlotinib | On-label | PR | 13.9 | 15.7 | 35.5 |
| 30 | 2 | *EGFR* p.(E746_A750del)  *EGFR* p.(T790M)  *EGFR* p.(C797S) | Brigatinib + cetuximab | No | No (unknown) | Brigatinib | Off-label |  |  |  |  |
| 31 | 2 | *EGFR* p.(E746_A750del)  *MET* amplification | EGFR TKI + crizotinib or EGFR TKI + capmatinib | No | Yes | Gefitinib +  crizotinib | Off-label | NE | n/a | 2.5 | 3.1 |
| 32 | 0 | *EGFR* p.(R776H)  *EGFR* p.(L858R) | Osimertinib | Yes | Yes | Osimertinib | On-label | PR | 28.1 (PFS1) | 30.8 (ongoing) | 30.8  (alive) |
| 33 | 2 | *EGFR* p.(E746_A750del)  *MET* c.(2942-30_2952del) (*MET* exon 14 skipping) | Osimertinib + crizotinib or osimertinib + capmatinib | No | Yes | Osimertinib +  crizotinib | Off-label | CR | 7.2 | 8.7 | 17.7 |
| 34 | 0 | *EGFR* p.(G719A)  *EGFR* p.(S768I) | Afatinib | No | Yes | Afatinib | On-label | PR | n/a | 1.8 (toxicity, switch to gefitinib) | 2.0 |
| 35 | 2 | *EGFR* p.(L747_A750delinsP)  *BRAF* p.(V600E) | Osimertinib + dabrafenib + trametinib | No | Yes | Dabrafenib + trametinib started | On-label | PD | 1.1 | 1.1 | 6.2 |
| 36 | 2 | *EGFR* p.(E709_T710delinsD) | Afatinib | No | Yes | Afatinib | On-label | NE (toxicity) | n/a | 2.5 (toxicity) | 5.7 |
| 37 | 0 | *EGFR* p.(E709_T810delinsD)  *PIK3CA* p.(H1047R) | Afatinib | Yes | Yes | Afatinib | On-label | NE (no evaluable lesions | 19.2 | 21.2 | 21.2 |
| 38 | 5 | *EGFR* p.(L747_T751del)  Loss of *EGFR* p.(T790M) | Rechallenge erlotinib | No | Yes | Erlotinib | On-label | PD | 2.5 | 3.5 | 19.5 |
| 39 | 2 | *EGFR* p.(E746_A750delinsIP)  *EGFR* p.(C797S) | Gefitinib or erlotinib | Yes | Yes | Erlotinib | On-label | PR | 9.4 | 10.4 | 36.9 |
| 40 | 3 | *EGFR* p.(E746_A750del)  *BRAF* p.(V600E) | Osimertinib + dabrafenib + trametinib | No | Yes | Osimertinib +  dabrafenib +  trametinib | On-label | PD | 1.5 | 2.2 | 6.4 |
| 41 | 1 | *EGFR* p.(S768I) | Afatinib (switch to afatinib) | ? | Yes | Afatinib | On-label | SD | 6.0 | 6.0 | 25.0 |
| 42 | 2 | *EGFR* p.(L858R)  *EGFR* p.(T790M) (very low allelic frequency)  *TP53* p.(R156C) (very low allelic frequency) | Osimertinib | No | Yes | Osimertinib | On-label | PD | 3.7 | 6.9 | 28.3 |
| 43 | 2 | *EGFR* p.(T751_I759delinsN)  *EGFR* p.(G724S)  Potential *RET* fusion (Nanostring positive, FISH *RET* negative) | Osimertinib + afatinib or afatinib | Yes | Yes | Afatinib | On-label | PD | 1.8 | 2.1 | 4.5 |
| 44 | 1 | *EGFR* p.(L747_P753delinsS)  *EGFR* p.(T790M)  *EGFR* p.(C797S) | Brigatinib (possibly in combination with cetuximab. Alternatively, chemo-immunotherapy) | Yes | Yes | Brigatinib | Off-label | PD | 1.6 | 1.6 | 31.0 (alive) |
| 45 | 1 | *EGFR* p.(S768I) | Afatinib | Yes | Yes, but treated <6 weeks | Afatinib | On-label | NE | n/a | 0.2 (clinical deterioration) | 2.2 |
| 46 | 2 | *EGFR* p.(L861Q)  *ERBB2* p.(S310F)  *EGFR* amplification | Afatinib | Yes | Yes, but treated <6 weeks | Afatinib | On-label | NE | n/a | 1.2  (toxicity) | 7.9 |
| 47 | 2 | *EGFR* p.(E746_A750del)  *EGFR* p.(T790M)  *KRAS* p.(G13D)  Loss of *RET* fusion | Osimertinib | No | Yes | Osimertinib | On-label | PD | 1.7 | 3.2 | 3.2 |
| ***ALK* fusion as (initial) driver mutation** | | | | | | | | | | | |
| 48 | 6 | *ALK* fusion  *ALK* p.(G1202R)  Loss of *ALK* p.(C1156Y) | Lorlatinib | Yes | Yes | Lorlatinib | On-label | PR | 3.9 | 4.9 | 12.3 |
| 49 | 4 | *EML4* exon 2::*ALK* exon 20 fusion  *MET* amplification | Crizotinib | No | Yes | Crizotinib | Off-label | PR | 5.4 | 6.4 | 9.8 |
| 50 | 2 | *ALK* fusion transcript, NOS  *ALK* p.(L1196Q) | Ceritinib | No | Yes | Ceritinib | On-label | PD | 2.8 | 5.1 | 9.2 |
| 51 | 5 | *ALK* fusion (without *ALK* resistance mutations) | Lorlatinib | No | Yes | Lorlatinib | On-label | SD | 28.3 | 29.5 | 36.6  (alive) |
| 52 | 2 | *EML4* exon 13::*ALK* exon 20 fusion  *ALK* p.(F1174C) | Alectinib or brigatinib | No | Yes | Alectinib | On-label | PR | 13.4 | 14.5 | 25.9  (alive) |
| 53 | 2 | ALK IHC+  NanoString negative. No resistance mutations. | Lorlatinib preferred, but only available after prior treatment with two other ALK inhibitors. Therefore, brigatinib or ceritinib, and lorlatinib afterwards. | No | Yes | Ceritinib | On-label | NE (toxicity) | n/a (toxicity) | 1.8  (toxicity, switch to lorlatinib) | 39.6  (alive) |
| 54 | 2 | *EML4* exon 2::*ALK* exon 20 fusion  *ALK* p.(F1174V)  Loss of *ALK* p.(L1196Q) | Lorlatinib | No | Yes | Lorlatinib | Off-label | CR | 12.3 | 13.8 | 28.1  (alive) |
| 55 | 1 | *EML4* exon 6::*ALK* exon 20 fusion  *ALK* p.(V1180L) | Ceritinib | No | Yes | Ceritinib | Off-label | PR | 7.3 | 8.2 | 26.3 |
| 56 | 4 | *ALK* fusion (no *ALK* resistance mutations) | Ceritinib | No | Yes | Ceritinib | On-label | PR | 2.9 | 5.0 | 5.2 |
| 57 | 1 | *ALK* fusion  *ALK* p.(C1156Y) | Alectinib | No | Yes | Alectinib | On-label | PR | 6.3 | 8.1 | 8.2 |
| 58 | 1 | *EML4* exon 6::*ALK* exon 20 fusion (no resistance mutations found) | Lorlatinib | No | Yes | Lorlatinib | On-label | SD | 13.3 | 16.8 | 16.8 |
| 59 | 4 | *EML4* exon 2::*ALK* exon 20 fusion (without *ALK* resistance mutations, mutation analysis performed five months earlier) | Lorlatinib | No | Yes | Lorlatinib | On-label | SD | 2.9 | 3.6 | 3.6 |
| 60 | 3 | *EML4* exon 6::*ALK* exon 20 fusion  *ALK* p.(G1202R) | Lorlatinib | No | Yes | Lorlatinib | On-label | PR | 21.2 | 24.2 | 34.0  (alive) |
| 61 | 2 | *ALK* fusion (additional molecular testing was not possible) | Lorlatinib | No | Yes | Lorlatinib | On-label | Unknown | 2.7 | 2.2 (due to other, concurrent terminal illness) | 2.7 |
| 62 | 1 | ALK IHC+  NanoString negative. | Alectinib at higher dose. Alternatively, switch to brigatinib. | No | Yes | Alectinib at higher dose | On-label | PD | 1.5 | 1.8 | 47.9  (alive) |
| 63 | 1 | *ALK* fusion (*EML4*_6a:*ALK*_20) (previously detected) (additional molecular testing was not possible) | Ceritinib | No | Yes | Ceritinib | Off-label | NE | 0.9 | 0.9 | 17.7 |
| 64 | 6 | *EML* exon 13::*ALK* exon 20 fusion (no resistance mutations found) | Rechallenge previous ALK-TKI | No | Yes | Alectinib | On-label | PD | 3.6 | 4.6 | 7.1 |
| ***BRAF* mutation as (initial) driver mutation(s)** | | | | | | | | | | | |
| 65 | 0 | *BRAF* p.(V600E)  *IDH1* p.(R132C) | BRAFi + MEKi | No | Yes | Dabrafenib + trametinib | On-label | PR | n/a | 4.1 (therapy switch, reason unknown) | 51.1  (alive) |
| 66 | 0 | *BRAF* p.(T599dup) | BRAFi + MEKi (with warning of potentially reduced efficacy), though standard treatment was preferred. | No | Yes | Dabrafenib +  trametinib | Off-label | PD | 1.8 | 2.2 | 32.4 |
| 67 | 0 | *BRAF* p.(V600K)  *MAP2K1* p.(Y130C) | BRAFi + MEKi | No | Yes | Dabrafenib +  trametinib | On-label | Unknown (no imaging evaluation, clinically good response) | n/a | 4.3 | 9.5 |
| 68 | 0 | *BRAF* p.(V600E)  *PIK3CA* p.(G1049R) | BRAFi + MEKi | No | Yes | Dabrafenib +  trametinib | On-label | SD | 4.0 | 5.3 | 14.9 |
| 69 | 0 | *BRAF* p.(V600E)  *MAP2K1* p.(L54P) | BRAFi + MEKi | No | Yes | Dabrafenib +  trametinib | On-label | NE (adjuvant setting) | n/a | 1.7 (toxicity) | 38.5 (alive) |
| 70 | 0 | *BRAF* p.(K601N) | Dabrafenib + trametinib | No | No (reason unknown) |  |  |  |  |  |  |
| 71 | 1 | *BRAF* p.(T599_V600delinsYSE) | Treat as if BRAF p.(V600E) | Yes | Yes | Dabrafenib + trametinib | Off-label | PR (neo-adjuvant setting) | 17.0 | 17.0 | 35.4 |
| 72 | 0 | *BRAF* p.(V600E)  *IDH1* p.(R132C) | BRAFi + MEKi | No | Yes | Dabrafenib +  trametinib | On-label | PR | 14.8 | 14.9 | 34.6 |
| ***MET* exon 14 skipping as (initial) driver mutation(s)** | | | | | | | | | | | |
| 73 | 0 | *MET* c.2913_2914delinsT (exon 14 skipping)  *PIK3CA* p.(H1047R)  *IDH2* p.(R140Q) | Crizotinib | No | Yes | Crizotinib | Off-label | PR |  | 1.9 | 1.9 |
| 74 | 0 | *MET* c.3028G>C (*MET* exon 14 skipping) | Crizotinib | No | Yes | Crizotinib | Off-label | NE |  | 0.9 | 0.9 |
| 75 | 5 | *MET* exon 14 skipping | Capmatinib | No | Yes | Capmatinib | Off-label | SD | 14.5 | 15.2 | 21.2 |
| 76 | 2 | *MET* c.3082G>C (*MET* exon 14 skipping) | Crizotinib or capmatinib | No | Yes | Crizotinib | Off-label | SD |  | 3.2  (treatment switch) | 5.3 |
| 77 | 0 | *MET* c.3072_3082+4del (*MET* exon 14 skipping) | Crizotinib or capmatinib | No | Yes | Crizotinib | Off-label | PR | 2.7 | 2.3  (toxicity + disease progression suspected) | 2.7 |
| **Other mutations as (initial) driver mutation(s)** | | | | | | | | | | | |
| 78 | 0 | IHC: ROS1 positive | Crizotinib | No | No (patient died shortly after MTB) |  | On-label |  |  |  |  |
| 79 | 0 | *KIT* p.(D816V) | KIT-targeted therapy | No | No (poor performance) |  | Off-label |  |  |  |  |
| L, number of prior systemic treatments (regardless of reason for discontinuation); MTB, molecular tumor board; BOR, best overall response; PFS, progression-free survival in months; DoT, duration of treatment in months; OS, overall survival in months; CR, complete response; PR, partial response; SD, stable disease; PD, progressive disease; NE, not evaluable; BRAFi, BRAF inhibitor; MEKi, MEK inhibitor. | | | | | | | | | | | |
